# Caught in the loop: the role of volitional control in the FoMO and social media addiction cycle

**DOI:** 10.3389/fpsyg.2025.1583921

**Published:** 2025-07-25

**Authors:** Yiheng Zhang

**Affiliations:** Krieger School of Arts and Sciences, Johns Hopkins University, Baltimore, MD, United States

**Keywords:** fear of missing out, social media addiction, volitional control, bidirectional relationship, digital behavior

## Abstract

This study explores the bidirectional relationship between fear of missing out (FoMO) and social media addiction, with particular attention to the mediating role of volitional control. A total of 621 participants completed an online survey incorporating three validated instruments: the Bergen Social Media Addiction Scale (α = 0.974), the Trait-State Misplaced Anxiety Scale (α = 0.951), and the Volitional Control Questionnaire (α = 0.709). Following data cleaning, 88.71% of responses were retained for analysis. Using SPSS 26.0 and AMOS, descriptive statistics, hierarchical regression, and mediation analyses were performed. The results demonstrate a robust positive association between FoMO and social media addiction. FoMO was found to influence social media addiction both directly and indirectly via volitional control, whereas social media addiction affected FoMO only through direct pathways. Notably, volitional control partially mediated the effect of FoMO on social media addiction but did not mediate the reverse relationship. These findings offer valuable theoretical insights into the dynamic interplay between FoMO and social media addiction and suggest that strengthening volitional control may be key to developing effective interventions targeting problematic social media use.

## 1 Introduction

The rapid advancement of smart devices and technology has led to the widespread use of various social media platforms, including WeChat, Douyin (Shake), and Kuaishou. While the growth of these platforms has significantly enhanced communication between people, it has also given rise to several negative consequences. More and more people are immersed in social media, and studies have shown that excessive use and attention can lead to behavioral addiction symptoms, referred to as social media addiction (Wang, 2023; Andreassen and Pallesen, 2014; Hawi and Samaha, 2017). Social media addiction not only affects real-life interpersonal interactions, life functioning, and work efficiency (Zivnuska et al., 2019), but also affects the development of an individual’s physical and mental health, such as: depression, self-esteem, sleep, and anxiety (Lin et al., 2016; Twenge and Campbell, 2019; Sherlock and Wagstaff, 2018; Van Der Schuur et al., 2019; Vannucci et al., 2017; Frost and Rickwood, 2017). That is why research on social media addiction is necessary. Some researchers have explored the influencing factors of social media addiction, which mainly include: intensity of social media use, immersion experience of social media use, fear of missing out, stress perception, online social support, offline social support, and education level (Dingle and Han, 2021).

Many previous studies have focused on adolescents or college students because they are often seen as high-risk groups for social media addiction. However, addictive use of social media is no longer limited to younger populations. Research has shown that adults of different age groups also experience problematic social media use and FoMO, especially as social networking platforms become more embedded in everyday life across generations (Andreassen et al., 2017; Yan, 2021). This study therefore includes participants from a wide range of ages and educational backgrounds, because it aims to capture a broader and more representative picture of how FoMO and social media addiction interact in the general population. This approach helps uncover patterns that may not be visible in studies focusing on narrow groups.

Previous research has investigated the relationship between social media addiction and fear of missing out (FoMO), presenting conflicting findings regarding the directionality of this relationship. Some research suggests that FoMO serves as a precursor to social media addiction, driving individuals to engage excessively with online platforms (Chai et al., 2018; Brown and Kuss, 2020). Conversely, other studies propose that social media addiction exacerbates feelings of FoMO, creating a cyclical pattern of compulsive behavior (Buglass et al., 2017; Hunt et al., 2018). The fear of missing out occurs when individuals worry that they are missing out on exciting or specific content (Chai et al., 2018; Vaughn, 2012). Previous studies have shown that about 66% of people have experienced this type of anxiety (Huguenel, 2017; Milyavskaya et al., 2018). That is why it is crucial to explore the relationship between social media addiction and fear of missing out.

Some relevant studies in China also have explored that the symptoms of FoMO can significantly predict social media addiction (Hu et al., 2022), and some researchers believe that social media addiction leads to fear of missing out (Chai et al., 2018), while others have found that the relationship between social media addiction and fear of missing out is bidirectional (Zhang et al., 2021). This shows that the relationship between social media addiction and fear of missing out is controversial, so this study further explores the direction of the relationship between the two, as well as the way of prediction between them, whether it is direct prediction, indirect prediction, or both. In the current study, this paper investigated the relationship between social media addiction and FOMO, and the mediating role of volitional control in the relationship between the two.

## 2 Literature review

### 2.1 Social media addiction and fear of missing out

Previous research has found that social media addiction is significantly correlated with negative emotions such as anxiety (Malaeb et al., 2021). Specifically, anxiety about missing out—often referred to as fear of missing out (FoMO)—has been identified as strongly associated with social media addiction (Al-Menayes, 2016). Studies have proposed three types of relationships between social media addiction and FoMO: First, FoMO can significantly predict social media addiction. For instance, Hu et al. (2022) investigated the relationship between missing out anxiety and social media addiction, finding that FoMO is a strong predictor of social media addiction. Similar findings were reported by Blackwell et al. (2017). Additionally, Li (2022) explored the relationship between missing out anxiety and social media addiction among middle school students and found that FoMO not only directly predicts social media addiction but also indirectly influences it. This suggests that the impact of FoMO on social media addiction may operate through both direct and indirect pathways. Thus, this study aims to further examine and validate the mechanisms through which FoMO influences social media addiction.

In contrast, scholars have also found cases where social media addiction leads to anxiety about missing out. For example, Buglass et al. (2017) found through a longitudinal study that the intensity of use of social media websites has a significant predictive effect on anxiety of missing out or fear of missing out 6 months later. When individuals are unable to use social platforms, anxiety levels are higher (Rosen et al., 2013a,b). There are few studies on the impact of social media addiction on FoMO. This study will explore whether social media addiction has an impact on missing-out anxiety and how it affects it, supplementing the research on the relationship between social media addiction and FoMO.

While most previous research has primarily focused on the unidirectional relationship, where fear of missing out (FoMO) predicts social media addiction. However, considering the mutual influence between social media addiction and anxiety about missing out, some studies have proposed a bidirectional relationship between these variables. For instance, Zhang et al. (2021) conducted a longitudinal study to explore the relationship between social media addiction and FoMO. The study found that participants’ initial social media addiction significantly predicted their levels of FoMO 8 months later. Conversely, initial levels of FoMO also significantly predicted social media addiction at the eight-month follow-up. Despite these findings, there remains a limited number of studies that have specifically examined this bidirectional relationship, which indicated a bland gap in the literature that requires further investigation.

### 2.2 Social media addiction and volitional control

Volitional control refers to an individual’s ability to elicit or maintain a disadvantageous response as well as plan execution by inhibiting his or her instinctive or advantageous response (Nigg, 2017), which is the core of individual self-regulation (Zhou et al., 2004). Individuals have to experience more and more things, responsible affairs, temptations, etc. as they grow older, then volitional control plays an important role in this process. As individuals age, they face increasing responsibilities, experiences, and temptations, and volitional control plays a crucial role in managing these challenges throughout the growth process. Some researchers used volitional control as a mediating variable to investigate the relationship between parenting styles and cell phone dependence in junior high school students (Lee, 2023), and the results showed that volitional control mediated the relationship between parenting styles and cell phone dependence, and that improving volitional control could reduce the degree of cell phone dependence. Teng and Zhang (2021) explored the effect of neuroticism on college students’ problematic mobile social network use, and used volitional control as a moderating variable, and the results showed that volitional control could modulate the effect of neuroticism on college students’ problematic mobile social network use. In summary, there is a correlation between volitional control and addictive behaviors such as cell phone dependence and problematic mobile social network use, and social media addiction is also a kind of addictive behaviors, therefore this present study took volitional control as a mediating variable to explore the relationship between Fear of Missing Out and social media addiction.

### 2.3 Fear of missing out and volitional control

Fewer studies have explored the relationship between volitional control and fear of missing out, and more have probed the relationship between volitional control and anxiety. For example, Mo et al. (2019) explored the mediating role of volitional control in the effect of parenting styles on adolescent children’s trait anxiety, and the results showed that volitional control partially mediated the relationship between parenting styles and children’s anxiety, which suggesting that volitional control has a certain effect on anxiety. Since the manifestation of anxiety of missing out is similar to that of anxiety, it is hypothesized that volitional control has an effect on FoMO.

In summary, previous studies have found both unidirectional and bidirectional relationships between social media addiction and fear of missing out, and it follows that the results of research on the relationship between the two variables are inconsistent. In the unidirectional relationship in which FoMO affects social media addiction, the influence of FoMO on social media addiction includes both direct and indirect ways, while the way in which social media addiction affects FoMO is not clear for the time being. Therefore, building on previous research, the present study used volitional control as a mediator variable to further investigate the relationship between social media addiction and fear of missing out and the mode of influence, so as to supplement the research on the relationship between social media addiction and fear of missing out, and to provide a direction for the research on the intervention of social media addiction. The research hypothesis is as follows: If a bidirectional relationship exists between social media addiction and fear of missing out, then volitional control serves as a partial mediator in this relationship.

This study offers a breakthrough perspective in understanding the psychological dynamics of social media use. Unlike prior research, which often examines FoMO and social media addiction as separate or unidirectional phenomena, this study investigates their bidirectional relationship and reveals how these variables may reinforce each other in a self-perpetuating cycle. Moreover, by introducing volitional control as a mediating factor, the study sheds light on a critical yet overlooked mechanism that could explain why individuals struggle to disengage from this cycle. To our knowledge, no prior study has simultaneously explored these interactions with a broad participant base. These insights not only fill a major gap in the literature but also provide actionable implications for developing interventions to curb social media addiction across diverse populations.

## 3 Methods

This study used a cross-sectional survey design to examine the bidirectional relationship between fear of missing out (FoMO) and social media addiction, as well as the mediating role of volitional control. Data were collected through an online questionnaire distributed to adult participants from diverse age groups and educational backgrounds. Standardized and validated scales were used to measure the core variables. The collected data were analyzed using descriptive statistics, correlation analysis, hierarchical regression, and mediation analysis in SPSS 26.0 and Amos.

### 3.1 Subjects

In this study, a survey method was employed to collect data. The scales were integrated into Questionnaire Star, an online survey tool, for data collection. A total of 700 responses were initially gathered. Responses that were deemed inconsistent or irrational (e.g., selecting the same option for all items) were excluded to ensure the data quality. After this screening process, 621 valid responses remained for analysis, and the validity rate of the data was 88.71%.

Subjects were selected by convenience sampling, leaving 621 subjects after invalid questionnaires were excluded (age: *M* = 33.26, SD = 9.29). Among them, 82% had a bachelor’s degree and 18% had a graduate degree. Duration of social media use: less than 30 min (22.8%), 30 min to 1 h (34.3%), 1–5 h (29.5%), and more than 5 h (13.3%). Purpose of social media use: keeping in touch with friends (28.3%), browsing news and current affairs (18.6%), sharing one’s own life and photos (11.3%), finding entertainment and diversion (10.5%), following celebrities and stars (9.8%), participating in social media groups and discussions (8.7%), and looking for business opportunities and partners (11.1%) (Table 1).

**Table 1:** Social media usage.

	Project	Percentage
Time of use	Less than 30 min	22.8%
	30 min to an hour	34.3%
	1–5 h	29.5%
	More than 5 h	13.3%
Purpose of use	Keeping in touch with friends	28.3%
	Browsing news and current events	18.6%
	Sharing their lives and photos	11.3%
	Looking for entertainment and diversion	10.5%
	Following celebrities and star-studded developments	9.8%
	Participating in social media groups and discussions	8.7%
	Seeking business opportunities and partners	11.1%

Data analyzed using SPSS 26.0.

### 3.2 Measuring tools

In order to assess the key variables in this study, three relevant validated scales from previous research were administered. These scales were carefully selected based on their relevance and established reliability in previous research.

#### 3.2.1 Social media addiction scale

The Bergen Social Media Addiction Scale (SNAS) (Andreassen et al., 2017), localized and validated by Liu (2021), was employed to measure social media addiction. The scale consists of 18 items rated on a 5-point Likert scale (1 = never; 5 = always). A higher total score indicates a greater level of social media addiction. In this study, the scale demonstrated excellent internal consistency with a Cronbach’s alpha coefficient of 0.974 (e.g., “You spend a lot of time thinking about social media or planning how to use it”).

#### 3.2.2 Trait–state fear of missing out scale

Fear of missing out was measured using the Trait–State Fear of Missing Out Scale developed by Wegmann and localized by Xiao and Liu (2019) The scale includes 11 items rated on a 5-point Likert scale (1 = not at all; 5 = fully). Since this study did not distinguish between trait and state anxiety, the total score was used, with higher scores indicating more severe fear of missing out. The scale’s reliability in this study was high, with a Cronbach’s alpha coefficient of 0.951 (e.g., “I fear that others have more rewarding experiences than me”).

#### 3.2.3 Volitional control scale

Volitional control was assessed using the 15-item Volitional Control Questionnaire (VCS) from the Revised Early Adolescent Temperament Problems (RETP), revised by Capaldi and Rothbart (1992). The VCQ items are rated on a 5-point Likert scale (1 = very poorly conformed; 5 = very well conformed), with higher scores indicating stronger volitional control. This scale has been validated for adult use (Teng and Zhang, 2021). In the present study, the Cronbach’s alpha coefficient was 0.709, indicating acceptable reliability (e.g., “I can resist doing things that I know are not good for me even if they feel tempting”).

### 3.3 Data analysis

Descriptive statistics, correlation analysis, and regression analysis were conducted using SPSS 26.0. Hierarchical regression analysis was conducted prior to mediation testing to examine the direct relationships between fear of missing out (FoMO), social media addiction, and volitional control. This approach allowed us to establish significant predictive effects and variance explained by each predictor before exploring potential indirect effects through mediation. By first determining the strength of these direct relationships, we could assess whether mediation analysis was methodologically justified.

For the mediation analysis, Amos was used to examine the mediating role of volitional control. Initially, fear of missing out (FoMO) was analyzed as the independent variable, social media addiction as the dependent variable, and volitional control as the mediator. The roles of FoMO and social media addiction were then reversed and analyzed again.

To better understand the bidirectional nature of FoMO and social media addiction, the roles of independent and dependent variables were swapped in subsequent analyses. This bidirectional testing captures the possibility that FoMO and social media addiction may reinforce each other over time, as suggested in prior literature (Zhang et al., 2021). This design also helps identify potential asymmetries in how volitional control mediates these relationships.

## 4 Results

### 4.1 Descriptive statistics

Descriptive statistical analysis was performed to summarize the data for each variable, with results presented in Table 2. The table provides the mean, standard deviation, maximum, and minimum values for fear of missing out, social media addiction, and volitional control.

**Table 2:** Descriptive statistics.

Variables	Mean	Standard deviation	Max	Min
Fear of missing out	32.49	11.65	55	12
Social media addiction	51.55	19.14	90	19
Volitional control	42.52	7.85	75	21

Data analyzed using SPSS 26.0.

### 4.2 Correlation analysis

Pearson’s correlation analyses were conducted to examine the relationships among FoMO, social media addiction, and volitional control. As Table 3 shows, it can be seen that there is a significant positive correlation between FoMO and social media addiction (*r* = 0.894, *p* < 0.001), indicating that higher levels of FoMO are associated with greater social media addiction. FoMO is significantly negatively correlated with volitional control (*r* = −0.391, *p* < 0.001), suggesting that as FoMO increases, volitional control decreases. Social media addiction is also significantly negatively correlated with volitional control (*r* = −0.430, *p* < 0.001), indicating that higher levels of social media addiction are associated with lower levels of volitional control.

**Table 3:** Correlation analysis.

Variables	Fear of missing out	Social media addiction	Volitional control
Fear of missing out	1		
Social media addiction	0.894***	1	
Volitional control	−0.391***	−0.430***	1

*p < 0.05, **p < 0.01, ***p < 0.001. Data analyzed using SPSS 26.0.

### 4.3 Hierarchical regression analysis

Hierarchical regression analysis was used to explore the relationship between FoMO and social media addiction. In the first model, FoMO was used as the independent variable, social media addiction as the dependent variable, and volitional control as the control variable in a regression analysis to examine the effect of FoMO on social media addiction. In the second model, social media addiction was used as the independent variable, FoMO as the dependent variable, and volitional control as the control variable in a regression analysis to investigate the effect of social media addiction on FoMO.

As the results shown in Table 4, FoMO exhibited a significant positive predictive effect on social media addiction (β = 1.471, *p* < 0.001), accounting for 80% of the variance. This result suggests that higher levels of FoMO are associated with greater social media addiction. After controlling for volitional control, FoMO remained a significant predictor of social media addiction (β = 1.420, *p* < 0.001), with an adjusted explanation rate of 80.8%. The 0.8% increase in explained variance suggests that volitional control partially mediates this relationship, however further analysis is required to clarify its specific mediating role. Volitional control showed a significant negative predictive effect on social media addiction (β = −0.232, *p* < 0.001), indicating that higher volitional control is associated with lower levels of social media addiction.

**Table 4:** Results of regression analysis.

The dependent variable	The independent variable	*R* ^2^	Adjustment *R*^2^	△*R*^2^	△*F*	β	*t*	*P*
Social media addiction	Step one	0.800	0.800	0.800	2477.594	3.768	3.696	<0.001
	Fear of missing out					1.471	49.775	<0.001
	Step two	0.808	0.807	0.008	24.577	15.602	6.028	<0.001
	Fear of missing out					1.410	44.731	<0.001
	Volitional control					−0.232	−4.958	<0.001
Fear of missing out	Step one	0.800	0.800	0.800	2477.594	4.444	7.396	0.000
	Social media addiction					0.544	49.775	0.000
	Step two	0.800	0.800	0.000	0.148	5.031	3.069	0.002
	Social media addiction					0.542	44.731	0.000
	Volitional control					−0.011	−0.384	0.701

Data analyzed using SPSS 26.0.

On the other hand, social media addiction significantly positively predicts fear of missing out (FoMO) (β = 0.544, *p* < 0.001), accounting for 80% of the variance. This indicates that higher levels of social media addiction are associated with increased FoMO. Even after controlling for volitional control, social media addiction remains a significant predictor of FoMO (β = 0.542, *p* < 0.001), with the explanation rate unchanged at 80%. This consistency suggests that social media addiction directly influences FoMO, independent of volitional control. Notably, volitional control does not significantly predict FoMO (β = −0.011, *p* = 0.701), which is reinforcing the conclusion that its role in this context is minimal.

### 4.4 Mediation analysis

**Figure 1:**
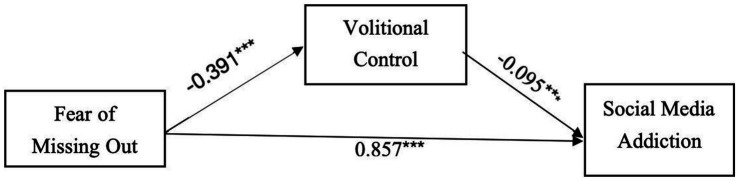
The mediating role of volitional control in the relationship between fear of missing out and social media addiction. ** refers to statistically significant paths in the model diagram.

A mediation analysis was conducted using fear of missing out (FoMO) as the independent variable, social media addiction as the dependent variable, and volitional control as the mediator. An observed variable mediation model was constructed, as all variables were explicitly measured. The model fit indices indicated a perfect fit with a Comparative Fit Index (CFI) of 1. The results of this mediation analysis are shown in Figure 1.

**Figure 2:**
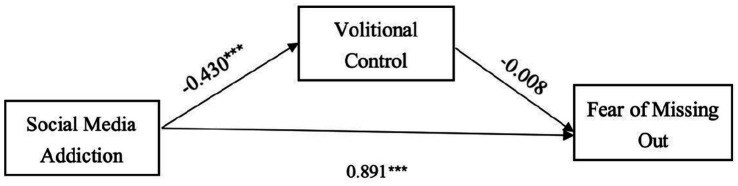
The mediating role of volitional control in the relationship between social media addiction and fear of missing out. ** refers to statistically significant paths in the model diagram.

The second mediation analysis was performed using social media addiction as the independent variable, FoMO as the dependent variable, and volitional control as the mediator. Similarly, an observed variable mediation model was constructed, achieving a CFI value of 1, indicating an excellent model fit. The results of this analysis are presented in Figure 2.

As shown in Figure 1, Tables 5, 6, FoMO has shown a significant direct predictive effect on social media addiction (β = 0.847, *p* < 0.001), indicating that higher levels of FoMO predict higher levels of social media addiction. FoMO also demonstrated a significant indirect predictive effect on social media addiction (β = 0.037, *p* < 0.001), with volitional control serving as a partial mediator (β = −0.095, *p* < 0.001). This suggests that FoMO can indirectly predict the degree of social media addiction through volitional control, confirming the partial mediating role of volitional control.

**Table 5:** Standardized weights of the mediating role of volitional control between FoMO and social media addiction.

Path	β	S. E.	C. R. (*z*)	*P*
Social media addiction ← fear of missing out	0.857	0.031	44.803	<0.001
Social media addiction ← volitional control	−0.095	0.047	−4.966	<0.001
Volitional control ← fear of missing out	−0.391	0.025	−10.587	<0.001
Fear of missing out ← social media addiction	0.891	0.012	44.803	
Fear of missing out ← volitional control	−0.008	0.029	−0.385	0.700
Volitional control ← social media addiction	−0.430	0.015	−11.875	

Data analyzed using SPSS 26.0.

**Table 6:** Standardized total, direct, and indirect effects of variables.

The dependent variable	The independent variable	Total effect	Direct effect	Indirect effects
Social media addiction	Fear of missing out	0.894***	0.857***	0.037***
	Volitional control	−0.095***	−0.095***	–
	Social media addiction	0.894***	0.891***	–
Fear of missing out	Volitional control	–	–	

Data analyzed using SPSS 26.0.

In contrast, as illustrated in Figure 2, Tables 5, 6, social media addiction had a significant direct predictive effect on FoMO (β = 0.891, *p* < 0.001), indicating that individuals with higher levels of social media addiction are more likely to experience heightened levels of FoMO. However, social media addiction did not show a significant indirect predictive effect on FoMO, and volitional control did not mediate the relationship between social media addiction and FoMO (β = −0.008, *p* = 0.700).

## 5 Discussion

This research explored the relationship between FoMO and social media addiction, with volitional control as a mediating variable. The study aimed to determine whether a bidirectional relationship exists between FoMO and social media addiction, and whether this relationship has direct effects, indirect effects, or both. The results indicated the following: (1) There is a significant positive correlation between FoMO and social media addiction, while both FoMO and social media addiction are significantly negatively correlated with volitional control; (2) FoMO has both a significant direct predictive effect and an indirect predictive effect on social media addiction; (3) Volitional control partially mediates the effect of FoMO on social media addiction; (4) Social media addiction only has a significant direct predictive effect on FoMO; and (5) Volitional control does not mediate the effect of social media addiction on FoMO.

The findings of this study reveal a psychological dynamic in which fear of missing out (FoMO) and social media addiction do not merely co-exist but appear to reinforce each other in a self-sustaining loop. Individuals with high FoMO are more likely to engage in compulsive social media use as a strategy to alleviate their anxiety about being excluded from rewarding experiences. However, this very reliance on social media may further heighten their sensitivity to what others are doing, which inevitably creates a vicious cycle of increased FoMO and deeper addiction. The regression results support this interpretation, showing that FoMO directly predicts social media addiction while also doing so indirectly through volitional control. This suggests that individuals who lack the ability to regulate their impulses are particularly vulnerable to allowing FoMO to dictate their online behaviors. On the other hand, when social media addiction predicts FoMO, the relationship is purely direct, with volitional control playing little mediating role. This asymmetry hints at a critical insight: while strengthening volitional control may help prevent FoMO from escalating into addiction, once addiction is established, breaking the cycle may require interventions that directly target compulsive behaviors and the emotional reliance on social media platforms.

### 5.1 The role of fear of missing out on social media addiction

Our findings that FoMO significantly predicts social media addiction are consistent with prior studies demonstrating this relationship (Blackwell et al., 2017; Hu et al., 2022). These studies suggest that individuals with high FoMO engage in excessive social media use as a way to alleviate anxiety about missing out. However, unlike Li (2022), who reported that FoMO fully mediated the pathway to addiction in adolescents, our data show only a partial mediation through volitional control. This difference may reflect cultural or age-group variations, as our sample included a broader adult population where regulatory mechanisms may function differently.

The results also show that FoMO predicts social media addiction in both direct and indirect ways, which illustrates that FoMO can not only directly predict social media addiction, but also indirectly predict social media addiction through volitional control. This result indicates that FoMO is a significant factor influencing social media addiction. When individuals are excessively concerned about missing out on important information or news related to others, their anxiety levels increase. If they attempt to alleviate this anxiety by constantly checking social media, it leads to increased social media usage, which can eventually result in social media addiction. Therefore, addressing social media addiction can be achieved by managing an individual’s FoMO. When the individual’s missing out anxiety decreases, the attention paid to information related to others will decrease, and they will not spend more time paying attention to the content of social media, thus improving the situation of social media addiction.

### 5.2 The role of social media addiction on fear of missing out

The results of the study showed that social media addiction showed a significant positive predictive effect on misplaced anxiety, which is consistent with previous research (Buglass et al., 2017; Bakioglu et al., 2022), indicating that when an individual has a high level of social media addiction, it can be predicted that the individual’s level of fear of missing out is higher. The result suggests that social media addiction is a factor that affects FoMO, and when an individual develops social media addiction, he or she will worry about missing some important news or missing some messages when he or she cannot access social media, which in turn leads to the strengthening of the level of missing out anxiety, so the individual’s FoMO can be alleviated by regulating the social media addiction or the use of social media. Therefore, when social media addiction decreases, the frequency of individuals’ contact with social media decreases, which gradually reduces their attention to news or other people’s information, and thus reduces their anxiety of missing out.

### 5.3 Bidirectional relationship between social media addiction and fear of missing out

The findings reveal a bidirectional relationship between social media addiction and FoMO, which is indicating that social media addiction significantly predicts FoMO, and in reverse, FoMO also significantly predicts social media addiction. It suggests that FoMO is a contributing factor to social media addiction, while social media addiction also influences the level of FoMO. However, there are differences in this bidirectional relationship. The effect of FoMO on social media addiction involves both direct and indirect pathways, whereas the influence of social media addiction on FoMO is only direct.

Several possible explanations for this result can be proposed. First, the higher correlation between the influences of social media addiction and FoMO plays a significant role in the relationship, while the lower correlation between other influences of FoMO and social media addiction does not, but this hypothesis needs to be further explored by using other influences as control variables. Second, volitional control as the chosen mediator, significantly predicts social media addiction but does not significantly predict FoMO, thus leading to differences in the bidirectional relationship between FoMO and social media addiction. In future research, certain variables that influence both social media addiction and FoMO as mediators could provide further insights into the relationship between FoMO and social media addiction.

### 5.4 The mediating role of volitional control in the relationship between social media addiction and FoMO

The mediating effect of volitional control in the path from FoMO to addiction aligns with Mo et al. (2019), who also identified self-regulatory capacity as a key buffer against anxiety-driven behaviors. The study results indicated that volitional control partially mediated the effect of FoMO on social media addiction. Specifically, while FoMO had a significant direct effect on social media addiction, it can also indirectly influence social media addiction through volitional control. This finding aligns with the study’s hypothesis and suggests that in addition to reducing FoMO, enhancing individuals’ volitional control could help alleviate social media addiction.

However, our finding that volitional control does not mediate the reverse pathway (addiction to FoMO) diverges from previous studies, such as Lee (2023), possibly due to differences in the conceptualization of regulatory functions across age groups. With the findings of mediation analysis considered, there are two potential explanations for this outcome. First, volitional control did not have a significant impact on FoMO. Second, the volitional control scale used in this study was a subscale of the Revised Early Adolescent Temperament Problems (RETP). Although validated for use in adults, it may not be the best measure for FoMO in a population with an average age of 30 years. Therefore, the scale might not have accurately captured the participants’ volitional control and caused a non-significant effect in this context.

### 5.5 Limitations and future research

Overall, these findings provide valuable insights for research and interventions targeting social media addiction and related online addictive behaviors. Although the results generally align with our research expectations, there are still some limitations to consider. One such limitation concerns the heterogeneity of the sample. Participants in this study came from diverse age groups and educational backgrounds, which reduced the homogeneity of the sample. While this heterogeneity may have introduced variability in how individuals experience FoMO and social media addiction, it also strengthens the generalizability of the findings across broader populations. As Yao et al. (2021) suggest, high heterogeneity allows study findings to be more broadly applicable to diverse populations, as the sample reflects a wider range of characteristics, backgrounds, and conditions found in the real world. Future studies could benefit from focusing on more homogeneous subgroups, such as college students or older adults, to explore whether the observed relationships hold within specific demographic contexts. Additionally, examining other potential mediators or moderators could further clarify the mechanisms linking FoMO and social media addiction.

Another limitation concerns the use of the Volitional Control Scale (VCS), which was originally developed as part of a temperament questionnaire for adolescents. Although this scale has been validated for use in adults in prior studies (Teng and Zhang, 2021), its conceptual focus and item phrasing may still reflect developmental contexts specific to younger populations. This raises the possibility that certain items may not fully capture the nuances of volitional control in older adults. Future studies could consider employing or developing a scale specifically designed for general adult populations to strengthen the validity of findings across diverse age groups.

### 5.6 Practical implications and policy recommendations

The present findings reveal a self-reinforcing loop between FoMO and social media addiction, in which individuals with low volitional control are particularly vulnerable to being caught in compulsive social media use. These insights suggest that interventions should not only address the emotional drivers of FoMO but also strengthen self-regulation capacities to help individuals disengage from addictive patterns.

At the individual level, cognitive-behavioral therapy (CBT) programs could be adapted to specifically target FoMO-related thought distortions, such as “If I do not check now, I’ll miss something important.” These programs may integrate volitional control exercises, such as practicing intentional delay in responding to notifications or scheduling fixed “offline windows” throughout the day. Prior studies have demonstrated that training in effortful self-control can significantly reduce compulsive technology use (Shanmugasundaram and Tamilarasu, 2023). Given that our study identified volitional control as a partial mediator between FoMO and social media addiction, interventions to strengthen this capacity may interrupt the cycle before it escalates. Platform-level design changes could complement these efforts. Social media companies could be encouraged to implement default usage limits or optional “low-stimulation modes” that hide non-essential updates and reduce FoMO triggers. As our results suggest, such interventions may be particularly helpful for users with low volitional control, which can help them to regain autonomy over their digital habits.

## Data Availability

The datasets presented in this study can be found in online repositories. The names of the repository/repositories and accession number(s) can be found in the article/supplementary material.
